# A Case Study of a Point-of-Care Electronic Medical Record [SABER] in Totonicapán, Guatemala: Benefits, Challenges, and Future Directions

**DOI:** 10.5334/aogh.3041

**Published:** 2020-09-24

**Authors:** Nicholas H. Aldredge, Dorian Rodriguez, Jessica González, David R. Burt

**Affiliations:** 1 University of Virginia School of Medicine, Charlottesville, VA, US; 2 UVA-Guatemala Initiative, Charlottesville, VA, US; 3 Xela, GT

## Abstract

**Background:**

The adoption of electronic medical records (EMRs) in lower-income nations has progressed slowly due to the lack of adequate infrastructure, funding, and training. However, EMRs have been successfully implemented previously in resource-limited health systems in South Africa, Haiti, Cameroon, Kenya, and Peru. Detailed, organized, and easily accessible medical records are particularly important in emergency departments due to the volume and acuity of the patient population.

**Methods:**

In order to further study the plausibility of an EMR in a resource-limited emergency department, a web-based, Spanish-language EMR known as SABER was developed for use in Hospital Nacional José Felipe Flores in Totonicapán, Guatemala. The software collects patient data including demographics, triage, initial evaluation, review of systems, physical exam, and evaluation and plan. It then generates a .pdf file consistent with information requirements of the Guatemalan Ministry of Health. Local physicians, medical students, and nurses were trained in the use of the software, which debuted in July 2016. To assess the effectiveness of SABER as an EMR, focus groups and Likert scale surveys were conducted with six physicians and 31 medical students working in the Hospital Nacional emergency department.

**Results:**

Thirty of 32 medical students and six of six doctors would recommend SABER to another provider. Positive aspects identified by staff include ease of use, quick data entry, and the potential for large data set research.

**Discussion:**

Remaining challenges include incorporating electronic nursing orders and lab results, troubleshooting technology problems including printer difficulties, a lack of electronic signature capability, and lack of integration with the rest of the hospital. Our study is consistent with other studies that show use of an EMR may help to reduce health disparities through improved patient records, medical data collection, and organization.

## 1. Introduction

### 1.1. Electronic Medical Record (EMR) Adoption in High-Income Countries

The potential benefits of electronic medical record systems (EMRs) in high-income countries have been well described. It has been suggested in the past that successful implementation may have a wide range of measurable benefits, including clinical decision support such as drug allergy warnings and drug incompatibilities [1], support for program monitoring including reporting outcomes, budgets, and supplies [2], support for clinical research, and management of chronic diseases such as diabetes, hypertension, and heart failure [3].

Additional studies in high-income countries have shown that EMRs have demonstrated significantly increased net monetary benefit per provider, cumulative net hospital savings, an increase in provider productivity, and improved provider efficiency [4,5,6]. A few studies have also shown evidence of improved clinical outcomes upon EMR adoption in high-income countries, including shorter hospital length of stay and lower 30-day mortality, a decrease in relative odds of death from myocardial infarction and CABG procedures, and improved outcomes for renal disease patients [7,8,9].

Despite the potential for many benefits, EMR adoption in high-income countries has also been associated with numerous drawbacks, including high cost of implementation and maintenance, changes to organizational workflow, temporary loss of productivity, and privacy and security concerns [10]. In addition, a 2014 survey of 6,375 American providers revealed that physicians who used EMRs had lower satisfaction with the amount of time spent on clerical tasks and higher rates of burnout [11]. Given these challenges and many others, it is important to the success of a given EMR to perform a detailed cost-benefit analysis.

### 1.2. EMR Expansion to Developing Countries

Given the potential benefits of EMR adoption demonstrated in high-income countries, efforts have been attempted to expand these systems to countries with limited resources. There are several examples of successful EMR and electronic health registry implementation in resource-limited hospitals in the current literature, including the Mosoriot Medical Record System in Kenya [12], PIH-EMR in Peru [13], HIV-EMR in Haiti, Careware in Uganda [14], the Baobab-ART (BART) system in Malawi [15], and an injury surveillance trauma registry in South Africa [16]. However, there is a general lack of studies that report on EMR systems that have been successfully implemented in hospitals in low- and middle-income countries (LMICs).

Despite successful implementation of the EMR systems described above, global adoption in resource-limited settings remains low. The reasons for this include: (1) resource and infrastructure limitations such as lack of reliable electricity, low-quality Internet access, and insufficient supply of devices; (2) lack of centralized organization such as a national health information technology (IT) agenda; (3) lack of explicit and broad legal regulation; (4) lack of common interoperability standards; and (5) lack of a trained workforce, including specialized IT workers and medical informaticians [17]. Additionally, EMR implementation represents a significant shift in workflow and organizational culture and thus may face significant barriers to adoption [18].

### 1.3. EMR Adoption in Guatemala

Guatemala is a country with poor health and economic indicators relative to the rest of Latin America and the rest of the world. Although Guatemala was reclassified by the World Bank in 2019 as an upper-middle-income country (UMIC) with a GNI per capita of US$4610, inequalities persist across geographic areas and among ethnic groups [19]. According to the most recent report in 2014 by the National Statistics Institute of Guatemala, 59.3% of the population of 16.58 million of Guatemala lived in poverty, and 8.7% lived below the lower-middle-income poverty line (defined as 8.3 in Guatemalan quetzal [2014] or US$5.5 per day in 2011 PPP) [20]. The GINI coefficient, a measure of inequality defined as the “extent to which the distribution among individuals or households within an economy deviates from a perfectly equal distribution,” was 0.43 as reported by the World Bank in 2014 and was consistent with moderate to severe economic inequality [19]. The life expectancy at birth was 72 years, compared to a life expectancy of 76 years for the associated World Health Organization (WHO) region [21].

Guatemala does not have a national universal health coverage policy or strategy, and it also lacks a national eHealth policy or strategy. EMR adoption in Latin America, including Guatemala, has been sparse, with only one example in the literature of an emergency surgery registry implemented in an urban hospital in Guatemala City [22]. However, after a one-year pilot in 2017 this registry was ultimately not adopted secondary to lack of optimization for hospital workflow, insufficient number of devices, and lack of end-user training.

Since 2015, the University of Virginia-Guatemala Initiative (UVA-GI) has worked on developing an EMR in a rural Guatemalan Hospital. Previous research groups identified Hospital Nacional Jose? Felipe Flores (Totonicapán Hospital) as a potential location for EMR implementation, and further researched what the required specifications for EMR software would be at this location [23,24]. Totonicapán Hospital was selected for the EMR system pilot because of its small size, a previously established relationship with UVA-GI, and willingness to collaborate on software optimization based on provider feedback. These features reduced the complexity and costs of implementation and increased the likelihood of maintaining a sustainable project.

Totonicapán is a city of 500,000 residents in the hot and humid western highlands of Guatemala, approximately 100 miles from the Guatemalan capital, Guatemala City. An overwhelming 97% of the population identify as indigenous peoples speak the K’iche’ language, although Spanish is also widely spoken. Of households in Totonicapán in 2014, 40% were classified as living in extreme poverty, as compared to a national figure of 23.4% [20]. Totonicapán has many prominent population health concerns including high infectious disease prevalence, high infant mortality, and a lack of medical personnel and resources. The mortality rate by the age of 15 is 13.5% and only 44.5% of the population reach the age of 65 [21]. Totonicapán Hospital contains approximately 94 beds, cares for roughly 2,000–5,000 patients annually, and employs 28 doctors [23].

### 1.4. Objectives

To address the stated needs of the Totonicapán Hospital Emergency Department (ED), UVA-GI developed and implemented a point of care EMR named SABER [Simple, Accesible, Básico, Electronic Record] beginning in 2015. Given the need for more research on the implementation, evaluation, and continuing support for EMRs in developing countries and particularly in Guatemala, this study had three main objectives within the context of a resource-limited environment:

1. Evaluate the perceived benefits of EMR implementation.
2. Identify specific and unique challenges to the implementation of an EMR in such a setting.
3. Identify potential strategies that Totonicapán Hospital could use to overcome these challenges.

## 2. Materials and Methods

This study was conducted with University of Virginia Institutional Review Board approval, protocol #2018-0256-00.

### 2.1. Software and Hardware Implementation

Implementation of the SABER program was conducted in multiple phases. First, we conducted a mixed-methods needs assessment with physicians, medical students, and hospital administration to determine the utility and feasibility of an EMR system in Totonicapán Hospital. Based upon the insight from previous research groups, an EMR, SABER 1.0, was developed in-house by two local Guatemalan UVA-GI programmers in 2015. The SABER interface is a Spanish language health record programmed primarily in PHP and Java that may be accessed via computers connected to a local area network (LAN) with a MySQL database. We chose to develop a novel EMR system as opposed to using an existing open-source platform (eg, OpenMRS, OpenEMR, GNUmed) because existing software was not easily customizable to unique needs of our hospital workflow, technological support for third-party programs was difficult to access internationally, and local computer engineers could offer on-site, in-person hardware and software support.

UVA-GI purchased a local Guatemalan internet connection for its program activities in the Totonicapán ED including cheap wireless routers to extend connectivity throughout the unit, but it was at that time not equipped to expand the internet service to the rest of the hospital or nearby clinics, laboratories, or other hospitals. Given these limitations, UVA-GI decided to pilot the SABER system exclusively in the Totonicapán Emergency Room. UVA-GI also purchased all of the necessary equipment to set up a fully functioning wireless LAN system, including a wireless LAN access point router, ethernet LAN switch, and ethernet cables and connectors. We also purchased a monthly subscription from a local internet service provider. Additionally, UVA-GI purchased an APC SMART-UPS 1000VA backup battery with the capability to provide up to 60 minutes of backup power for the system, a Canon iP2800 printer, a double-sided Canon laser printer, ink cartridges (color included), and multiple reams of paper. The total cost of these hardware components including internet service for one year was $1,584.

SABER collects data, including basic patient information, triage, initial evaluation, review of systems, physical exam, and evaluation and plan. It generates a PDF file based upon data entered into the browser form. The information collected is consistent with the patient information that is required to be reported to the Guatemalan Ministry of Health (GMH). Subsequent versions of SABER after the v1.0 pilot eliminated the need for filling out a paper chart in addition to inputting information in the EMR. These versions also addressed various bug fixes and expanded the capacity of the EMR to include order input. In the latest version of SABER (v. 6.0), all patient information stored electronically, including medication orders, can be printed directly for reporting to GMH. The GMH does not currently have capacity to accept or process data electronically, so printing capability is a critical feature of SABER.

### 2.2. Survey Development

In order to assess the continuing viability of SABER with regards to efficient collection of patient data and provider perceptions of the EMR system, we created a survey for medical students and emergency department healthcare providers. The survey was developed by the investigators, with guidance from the preliminary results of investigations conducted in prior years in the Totonicapán Hospital ED. We also consulted with other members of the UVA-GI team including healthcare professionals who had worked in Guatemala, in-country administrative professionals who work with the Totonicapán Hospital administration, and individuals involved in the networking and implementation of the SABER system. The survey was also informed by a systematic review of previous surveys that focused on electronic medical record adoption [25,26].

The survey included questions designed to elicit basic demographic information, prior experience using an EMR, and Likert surveys evaluating providers’ perception of and experiences with the SABER EMR. A 5-point Likert survey was used to evaluate provider perceptions of the SABER EMR, whereas a 7-point survey was used to evaluate their self-reported proficiency with the program in order to better distinguish differences among computer skills.

The survey was administered in Spanish to 31 medical students on their clinical rotations between fourth and sixth year, and to six physicians in the Totonicapán ED. The surveys were given to the medical director of the ED, who subsequently distributed the surveys to the participants in-person. Participation in the survey was completely voluntary, and participants were instructed that they could withdraw their responses at any time. No personal identifying information was collected through the survey; participants were assigned a unique, randomized personal identification number. There was a total of 40 medical students and eight doctors working in the emergency department in 2018, thus yielding a survey participation of 80% and 75%, respectively. No participants declined to participate in the survey; participation was limited by providers who were off-service at the time of the investigation. Characteristics of survey participants are outlined in Table 1.

**Table 1:** Characteristics of Survey Respondents and Their Practices.

Characteristic	Respondents (n = 37)

***Physicians***	n = 6
*Sex*	
M	4
F	2
*Years of medical practice*	
1–9	4
10–19	1
20+	1
*Completed residency?*	
Y	3
N	3
*Physician specialty*	
Primary care	2
Not primary care	3
Unspecified	1
*Time working in Totonicapán Emergency Room*	
< 1 year	2
1–4 years	2
5–9 years	–
10+ years	2
*Used EMR previously*	
Y	4
N	2
*Time using SABER (months)*	
0–5	4
6–12	1
24+	0
Data not collected	1
***Medical Students***	n = 31
*Sex*	
M	10
F	21
*Year in medical school*	
4th	19
5th	2
6th	10
*Medical school attended*	
Universidad Mesoamericana	26
Universidad de San Carlos	3
Not specified	2
*Used EMR previously*	
Y	1
N	30
*Time using SABER (months)*	
0–5	23
6–12	3
13+	4
Data not collected	1

### 2.3. Qualitative Evaluation Process

In addition to surveys of healthcare providers in the Totonicapán Hospital Emergency Room, we also conducted a series of four focus group interviews in Spanish consisting of 4–6 individuals each of either physicians or medical students. Focus group interviews were conducted using a series of 10 scripted open-ended questions and lasted approximately 30–60 minutes. These interviews were facilitated by a medical student fluent in Spanish without prior relationship to the participants (the first author). Before taking part in focus group discussions, participants were informed of the purpose of the study and that their participation was completely voluntary and that their responses would be anonymous. Confidentiality and privacy of the participants were ensured. All focus groups were proctored by a professional transcriptionist who documented the general content of the discussion.

Initial analysis of focus group data was performed by the same individual who had conducted the interviews. The goal of the analysis was to identify themes related to the original objectives of the study. Attention was given to perceived benefits of and challenges to the SABER EMR, and common topics and/or challenges addressed by participants were given priority. The researcher who conducted the interviews engaged in ongoing discussion with other members of the research team, the director of the Totonicapán ED, and developers of the SABER program. Themes selected were: (1) organizational workflow; (2) critical technical system features; and (3) social and cultural system features that either helped or hindered EMR adoption. The results of both the survey and focus group interviews were deemed as critical to understanding the appropriate organizational issues, correct system features, and workplace culture needed to create an EMR that may be successfully adopted in a resource-limited setting.

## 3. Results

### 3.1. Survey Results

Results of the survey as described in Tables 2 and 3 are summarized below.

**Table 2:** Survey Results of Provider Perceptions of SABER.

Survey response *Medical students (n = 31)*	1	2	3	4	5

**Quality of continuing service and support for SABER**					
UVA-GI provides continuous appropriate support in order to use SABER effectively.	0	3 (10%)	2 (7%)	12 (39%)	14 (45%)
UVA-GI helps with the training necessary to use SABER.	3 (10%)	1 (3%)	4 (13%)	17 (55%)	7 (23%)
**Overall satisfaction with SABER**					
If I had the opportunity to select another EHR, I would choose SABER.	0	0	4 (13%)	20 (65%)	7 (23%)
SABER improves the quality of care in the emergency room in Totonicapán.	0	0	9 (29%)	17 (55%)	5 (16%)
I would recommend SABER to another healthcare provider.	0	0	1 (3%)	16 (52%)	14 (45%)
If I could return to paper-based medical records, I would do so	7 (23%)	10 (32%)	8 (26%)	4 (13%)	2 (7%)
**Survey response** ***Physicians (n = 6)***	**1**	**2**	**3**	**4**	**5**

**Quality of continuing service and support for SABER**					
UVaGI provides continuous appropriate support in order to use SABER effectively.	0	1 (17%)	0	2 (33%)	3 (50%)
UVaGI helps with the training necessary to use SABER.	0	1 (17%)	0	2 (33%)	3 (50%)
**Overall satisfaction with SABER**					
If I had the opportunity to select another EHR, I would choose SABER.	0	0	0	3 (50%)	3 (50%)
SABER improves the quality of care in the emergency room in Totonicapán.	0	0	0	4 (67%)	2 (33%)
I would recommend SABER to another healthcare provider.	0	0	0	3 (50%)	3 (50%)
If I could return to paper-based medical records, I would do so	3 (50%)	0	1 (17%)	1 (17%)	1 (17%)

**Table 3:** Survey Results of Participant Skills Using SABER.

Survey response *Medical students (n = 31)*	1	2	3	4	5	6	7

In general, I have sufficient experience using a computer	0	0	1 (3%)	5 (16%)	2 (7%)	4 (13%)	17 (55%)
In general, I have sufficient experience using SABER	1 (3%)	1 (3%)	1 (3%)	7 (23%)	7 (23%)	7 (23%)	7 (23%)
I know how to print a chart using SABER	1 (3%)	1 (3%)	0	0	0	5 (17%)	24 (77%)
I know how to admit a patient using SABER	3 (10%)	0	0	1 (3%)	0	4 (13%)	22 (71%)
I always user SABER to input patient information in the Totonicapán Emergency Room	2 (7%)	0	0	3 (10%)	3 (10%)	6 (19%)	17 (55%)
**Survey response** ***Physicians (n = 6)***							

In general, I have sufficient experience using a computer	0	0	0	0	0	0	100
In general, I have sufficient experience using SABER	1 (17%)	0	0	0	1 (17%)	2 (33%)	2 (33%)
I know how to print a chart using SABER	1 (17%)	0	0	0	1 (17%)	2 (33%)	2 (33%)
I know how to admit a patient using SABER	1 (17%)	0	0	0	0	2 (33%)	3 (50%)
I always user SABER to input patient information in the Totonicapán Emergency Room	1 (17%)	0	0	0	0	1 (17%)	4 (66%)

#### 3.1.1. Quality of continuing service and support for SABER

The quality of continuing service and support for SABER was reported to be appropriate by medical students, with 26 out of 31 (84%) agreeing or strongly agreeing that UVA-GI provided continuous and appropriate support to use SABER, and 24 out of 31 (78%) agreeing or strongly agreeing that UVA-GI provided the training necessary to use SABER. Four out of six (66%) doctors agreed or strongly agreed that the support provided by UVA-GI for SABER was appropriate.

#### 3.1.2. Overall satisfaction with SABER

In general, participants appeared to be satisfied with the use of SABER in the hospital, including both medical students and physicians. Thirty of 32 (94%) medical students and six of six doctors said they would recommend the use of SABER to another healthcare provider. Zero medical students or physicians reported on the survey that they were unsatisfied with the SABER system.

In response to the question, “If I could return to paper-based medical records, I would do so,” participants’ responses were divided between “No” or “Neutral.” The majority of medical students (32%) responded to the question with “somewhat disagree,” but 26% of medical students stated that they were unsure of their response to the question. For physicians, three out of six (50%) said that they strongly disagreed with the statement. The rest were evenly divided between unsure and affirmative responses.

#### 3.1.3. Computer skills

Self-reported responses to the survey generally indicated that participants felt comfortable using a computer. Medical students reported knowing how to use specific functions of SABER to carry out specific tasks as printing a chart or admitting a patient. Four out of six (66%) doctors who responded to our survey reported that they had sufficient experience using SABER and carrying out specific functions related to SABER.

### 3.2. Focus Group Results

Focus group results were organized into the three themes of organizational workflow, critical technical system features, and social and cultural system features as described previously.

#### 3.2.1. Organizational workflow

The Totonicapán ED is relatively small, with six dedicated adult beds, a single trauma bay equipped with two beds and a ventilator, and a smaller area with two beds for pediatric and neonatal patients. There is a waiting room in the front of the emergency department with a triage desk that is staffed by a secretary from 7:30am to 4:30pm Monday through Friday. Patients are tracked in the waiting room on a registration sheet; SABER is not yet programmed with an integrated triage system. When patients are brought back to one of the beds in the department, a provider (typically a medical student) enters patient information into SABER on one of seven laptop computers provided by UVA-GI. Once completed, the chart is printed in the front of the ED and signed/stamped by a physician. Completed charts are saved as they must be reported to the GMH.

Participants of both student and physician groups identified the following positive organizational workflow aspects of SABER: (1) faster data entry, especially in recent versions which avoid double data entry into both paper and electronic charts; (2) elimination of the problem of lost medical records; and (3) increased data access for clinicians, administrators, and for legal matters. Challenges to organizational workflow included lack of clearly defined professional roles for data transcription, lack of integrated emergency room and laboratory computer systems, lack of electronic nursing order functionality, and lack of integration with the rest of the hospital medical records resulting in incomplete patient data.

#### 3.2.2. Critical technical system features

Focus group participants were asked to identify key technical features of SABER that were either helpful or detrimental to the functioning of the Totonicapán ED. Helpful features of SABER included increased legibility versus paper records resulting in improved inter-provider communication and increased control and analysis of systems-level hospital data. Technical challenges associated with SABER included unreliable power supply resulting in intermittent LAN outages, lack of an electronic signature function thus requiring providers to sign printed paper copies of charts, hardware malfunction (most frequently, printer errors), and software troubleshooting problems requiring 24-hour technical support.

#### 3.2.3. Social and cultural system features

The initial implementation process of SABER in 2015 was developed based on feedback from key hospital stakeholders including hospital administration, medical directors, physicians, nursing staff, and medical students. Since then, UVA-GI has continued to provide on-site technical support and training for end users, with software developers on-site during the week and available by phone at night and on the weekends. Implementation was conducted in stepwise fashion with the goal of accommodating staff to the use of SABER before using the software with all the providers in the ED. At the time of this investigation the most recent version of SABER (v6.0) was in the process of adaptation for use with nursing staff.

Focus group participants noted lack of consistency among stakeholders as a key aspect preventing further successful expansion of SABER. For example, although UVA-GI spent several months piloting a new software version with integrated nursing orders that included hands-on training and one-to-one support, some nursing staff still refused to use SABER and continued to handwrite orders. Participants suggested that one possible reason for this refusal was a relative lack of technical proficiency among nursing staff, even though they had received dedicated training in the use of SABER for several months. In addition, some medical students reported that there were several physicians who would refuse to enter patient information using SABER.

## 4. Discussion

### 4.1. Lessons Learned

The majority of participants had a favorable impression of SABER and seemed to indicate an understanding of why an EMR adoption could have a positive impact on both patient care and ED organization and workflow. However, we found that achieving these positive changes would require a shift in workplace culture and workflow not previously identified as a challenge. Unfortunately, nurses were not included as participants in the investigation because they were not end users at the time of study design. We learned during our investigation that future investigations must include nurses as key stakeholders to the future success of SABER. Their lack of inclusion in this study may have unfortunately amplified already existing political and cultural tensions among providers in the Totonicapán ED.

We also learned that efficient and effective hospital workflow is important in maintaining accurate medical records and continued use of an EMR. Without clearly defined roles for data input, oftentimes patient data was not collected in a timely fashion resulting in potential loss of information. Additionally, without a clearly defined triage system patients could end up waiting up to two hours to receive treatment and were often not identified as patients in the waiting room. This is a potential future application of SABER.

### 4.2. Recommendations

Two studies describing methods used to overcome social barriers to EMR adoption in developing countries appeared useful as analogues to the challenges faced by SABER. The first was a 2010 case study of an EMR system at a large hospital in India that used financial and social incentives to encourage chart completion using the EMR as opposed to the old method using paper charts. Use of the EMR was kept optional in order to reduce pressure on staff, but providers who chose to use the EMR received a small bonus as well as recognition in the EMR system via a “top 10” list. These incentives increased user participation without the need for administrative coercion [27].

A second cross-sectional study conducted in 2014 in six public and private hospitals in Saudi Arabia identified resistance to using new technology as a key social barrier obstructing EMR implementation. Importantly, the study results showed that 70% of excluded survey responses due to returned questionnaires were from nurses, indicating a relative lack of participation in the EMR adoption process. However, the authors of this study argued that nurses are “critical stakeholders who can affect EMR implementation either positively or negatively, and [that] significant attention should be directed to them in [EMR] adoption [28].” The authors found two key solutions to addressing user resistance. First was an adequate, strong, committed, and positive leadership team including engineers and training supervisors [29]. The second solution was conducting education training sessions to instruct the users [30]. These solutions are also applicable to the needs of the Totonicapán ED.

Based on the barriers to EMR implementation identified from survey responses, focus group discussions, and observation of delivery of care in the Totonicapán Hospital Emergency Room, we categorized our recommendations for future implementation of EMRs in low-resource settings into seven categories which had previously been identified as success criteria in such a setting in Table 4 [31]. Based on our observation that there was significant organizational and political resistance from providers in adopting the SABER electronic medical record, we suggest that these barriers to adoption be addressed first, before then addressing additional important technical, financial, ethical, functionality, and training barriers. Organizational and user resistance to EMR adoption in the Totonicapán ED appear to be mediating factors on other barriers.

**Table 4:** Recommendations for Implementation of EMRs in Low-Resource Settings.

Identified Barriers	Suggested Solutions

***Organizational:** Focuses on (managerial) circumstances within the organization itself, e.g. human resources, adequate support staff, attitude toward EMR, stakeholder involvement*	
Healthcare professionals are not motivated to adopt the EMR system	– Increase participation in EMR implementation in specific phases – Provide adequate and ongoing professional training – Ensure robust leadership that encourages all providers to use the EMR – Consider making EMR use voluntary with financial and social incentives
Staff do not understand the importance of the data collected by EMRs	– Hold frequent department-wide meetings to discuss data collected from the EMR and to create data-driven goals for future preventative care measures
Lack of managerial support/leadership for EMR implementation	– Hold frequent meetings with hospital leaders to provide adequate technical support and encourage leaders to promote EMR adoption within the culture of their hospital unit – Ensure that management provides effective monitoring of appropriate EMR use by hospital staff
Workflow of the unit/hospital does not match the design of the EMR	– Adjust the workflow of both the unit/hospital and EMR to ensure seamless integration. For example: Place workstations at each patient bed to ensure information is entered in a timely fashion Encourage specific procedures that include EMR use at the time of patient care Draw out map of hospital workflow; customize EMR to match hospital workflow

***Political:** Trust, attitude to change, general political willingness, includes legal health policies and country-wide circumstances including hospital location*	
Resistance from providers who have not used EMRs in the past	– Provide adequate and ongoing training to providers before they are required to use the EMR – Hold meetings with providers resistant to change to understand why they do not wish to adopt the EMR
Lack of communication between different healthcare providers within the unit or hospital	– Hold interdisciplinary department-wide meetings to discuss the integration of EMR systems among different professional
Lack of provider trust in data collected by EMR system; fear of accountability	– Discuss with providers the improvement in quality of care through the reduction of medical errors by EMR data collection and analysis

***Functionality:** System architecture and functions, e.g. data handling in different forms, having a system dictionary, usability*	
EMRs add additional tasks that take longer to complete	– Eliminate unnecessary and redundant data input into the EMR system – Include a system dictionary with algorithms allowing user-shortcuts for data entry
EMR system requires “double work”, meaning that providers fill out the same information twice both electronically and on paper	– Eliminate redundancy between paper and electronic forms – Design a completely digital system that may be easily printed out if records are needed for reporting to government agencies

***Training:** Training using the system, handover to local support staff, educational background and knowledge such as computer literacy of staff*	
Vulnerability of program to loss of critical programmer	– Coach and train additional programmers who understand the electronic medical record – Develop EMR on open-source platform
Lack of around-the-clock technical support	– Establish hours for phone support manned by program administrators – Train participants to troubleshoot common hardware and software problems – Create technical handbook with step-by-step solutions to common EMR problems – Hire additional technical support staff to provide additional hours of in-person support
Limited technical proficiency of end-users	– Implement a basic technology professional education program to ensure sufficient technical capacity to use computers and software – Provide adequate and ongoing support to all potential users of the EMR system

***Technical:** Includes infrastructure, e.g. internet access, power supply, software architectural characteristics, security and privacy*	
Power supply is unreliable	– Install a backup power supply with surge protection for EMR equipment, such as a generator system or series of batteries – Consider alternative power supply (e.g. solar)
Troubleshooting problems, including both hardware and software	– Create a laminated technical handbook with the most commonly identified troubleshooting problems and a step-by-step solution; place strategically around hospital; mirror this print edition with a digital backup

***Financial:** Availability of resources and funding, efficiency*	
Lack of available dedicated EMR support staff	– Train hospital staff to troubleshoot common problems, minimizing reliance on support staff

***Ethical:** Sustainability, privacy and security, regulatory and cultural issues*	
The EMR is dependent on technical and financial contributions from outside stakeholders	– The sustainability of a project is heavily dependent on effective leadership and training of the involved stakeholders in the target population. – After start-up costs have been covered and ongoing costs have stabilized, slowly transition more financial responsibility onto the receiving institution.

### 4.3. Study Limitations

Given the size of the Totonicapán Emergency Department, we were faced with a limited sample size for our research given the small population size. For example, we only collected quantitative survey data from six doctors, which limited further statistical analysis of their responses. However, by collecting qualitative data from the participants we elicited valuable information regarding the continuing efficacy of SABER even with the limitations of our data analysis.

As previously mentioned, our investigation did not include nurses as participants as they were not end-users at the time the investigation was planned. However, adding nursing order functionality and encouraging nurses to use SABER would require input from nurses to best design a program that meets their unique needs. A successful EMR requires nursing feedback, investment, and participation [28]. Consequently, expansions to other units in the hospital will require similar input from future stakeholders.

We also recognize that SABER is an EMR in a very specific setting with a unique user interface, support structure, and patient demographic. Therefore, we caution against the generalizability of the results of this study when compared to EMR implementation in other care settings and/or countries. The hope is that this study provides information for others hoping to implement EMRs in low-resource settings, but not direct replication of our system in other settings without consideration of local contexts.

### 4.4. Future directions

We have several initiatives and research opportunities for future consideration that we argue would improve both quality of care and quality of data in the Totonicapán ED, including (1) expansion of SABER to include pharmacy data, (2) increasing provider education through development of an online and in-person laminated troubleshooting handbook, (3) integration with waiting room triage procedure, (4) correlation of SABER metadata to patient outcomes, and (5) potential for mobile health expansion. With these future initiatives, we hope to successfully expand SABER within the Totonicapán Emergency Department and eventually to the rest of the hospital, with the goal of providing a model for successful implementation of an EMR in a resource-limited setting.

## Author’s Information

**Nico Aldredge**

Mr. Aldredge is a fourth-year medical student at the University of Virginia School of Medicine and was awarded a $4,000 research grant from the University of Virginia Center for Global Health to conduct this investigation. He had full access to all the data in this study and takes complete responsibility for the integrity of the data and the accuracy of the data analysis.

**Dorian Rodriguez**

Mr. Rodriguez is a Guatemalan computer scientist who helped program the initial version of SABER in 2015 and has been a part of continued development since then. He receives funding for his work from the UVA-Guatemala Initiative.

**Jessica González**

Ms. González was one of the co-founders of the UVA-Guatemala Initiative along with Dr. David Burt. She performs an administrative role as the in-country program director. She is paid for this role by the UVA-Guatemala Initiative.

**David Burt, MD**

Dr. David Burt founded the UVA-Guatemala Initiative along with Jessica González. He is an emergency medicine physician and associate professor of emergency medicine at the University of Virginia.
